# Up Close with Ticks

**DOI:** 10.3201/eid2901.AC2901

**Published:** 2023-01

**Authors:** Byron Breedlove

**Affiliations:** Centers for Disease Control and Prevention, Atlanta, Georgia, USA

**Keywords:** art science connection, emerging infectious diseases, art and medicine, about the cover, George Marx, vector-borne diseases, ticks, illustration of ticks, (Ixodida), bacteria, rickettsia, parasites, up close with ticks, Rocky Mountain spotted fever, Howard T. Ricketts, Rickettsia rickettsia, Lyme disease, tickborne relapsing fever, Crimean-Congo hemorrhagic fever, Heartland virus disease, Bourbon virus disease, human granulocytic anaplasmosis, Kyasanur Forest disease, human monocytic anaplasmosis, tick-borne encephalitis, Powassan encephalitis, babesiosis, theileriosis, ehrlichiosis, pathogenic microorganisms, zoonoses

**Figure:**
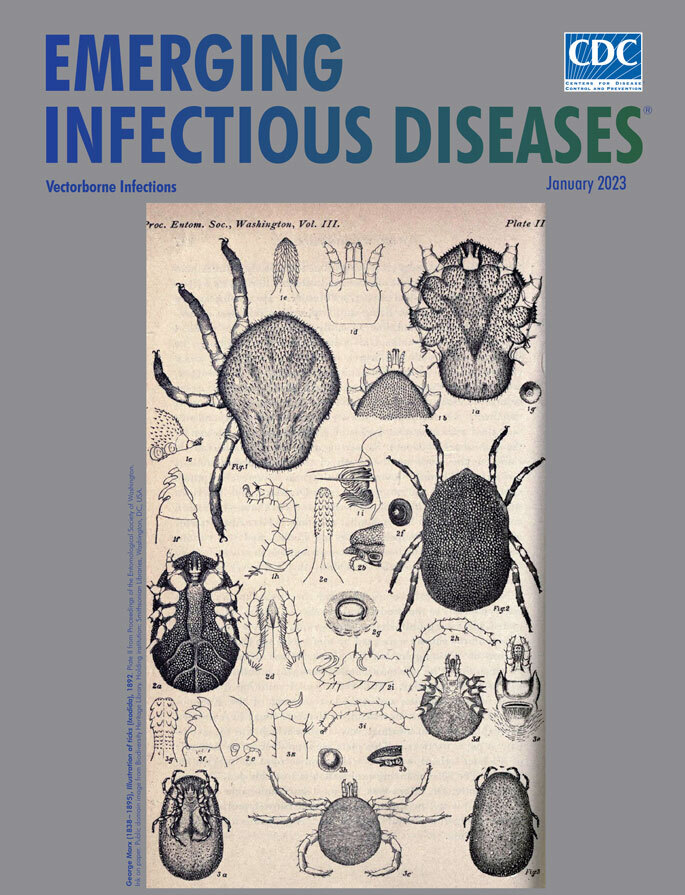
**George Marx (1838−1895), *Illustration of ticks* (*Ixodida*), 1892.** Plate II from Proceedings of the Entomological Society of Washington. Ink on paper. Public domain image from Biodiversity Heritage Library. Holding institution: Smithsonian Libraries, Washington, DC, USA.

Worldwide, only mosquitoes spread more vectorborne diseases than ticks; however, in temperate areas of North America, Europe, and Asia, ticks cause most vectorborne diseases. Ticks are vectors for multiple viruses, bacteria, and parasites that cause can cause an array of infectious diseases. Researcher Daniel Sonenshine notes, “Ticks transmit a greater variety of pathogenic microorganisms than any other hematophagous arthropod.” 

Ticks were spreading pathogens for millions of years before humans evolved. Biologist George Poinar, Jr., has found evidence of *Borrelia,* a type of spirochete-like bacteria that causes Lyme disease, in fossilized ticks preserved in 5−20-million-year-old amber. Much older fossilized ticks have been found in 99-million-year-old amber. However, that ticks are vectors for infectious diseases that affect humans was not confirmed until the early 20th Century.

Recently, these tiny arthropods gained additional notoriety by purportedly being the first living animals to be filmed under a scanning electron microscope. Science journalist James Gorman offers this colorful account: “Chain saws, hockey masks and the undead are all classic symbols of horror. But for a true shiver of dread, take a look at a tick. When seen with an electron microscope, a tick’s mouth has what look like twin saws (chelicerae) flanking an appendage (a hypostome) that appears to be the kind of long, barbed sword that a villain in a video game might favor.” 

Images and videos from such research reveal new insights into how ticks penetrate and remain attached to hosts and transmit pathogens. They also confirm that some hand drawn illustrations of ticks from the late 19th century were remarkably detailed and accurate. This month’s cover image, *Illustration of ticks (Ixodida)*, by George Marx, is an excellent example. 

Born and educated in Germany, Marx enrolled in the gymnasium at Darmstadt in the Hesse district when he was 14 years old. His obituary notes that while he was a student there, Marx “proved himself so proficient in botany, and at the same time so able an artist, that to him was assigned the task of making the illustrations for the Flora of Gross-Gerua,” the district seat in that part of Germany. 

After earning his degree in pharmacy in 1860, Marx left Germany for the United States, where he served in the American Civil War, receiving an honorable discharge after experiencing a serious wound and illness. In 1865, Marx moved to Philadelphia, where he began collecting Arachnida. In 1878, Marx relocated to Washington, DC, after accepting a position as a natural history illustrator in the Division of Entomology, US Department of Agriculture. Cited for his meticulous artwork, in 1889, Marx was selected to be chief of the department’s newly established Division of Illustrations, where he worked and devoted much of his time to studying ticks until just before his death in early 1895. A charter member of the Entomological Society of Washington, DC, Marx served as its fourth president. His obituary notes “. . . the various plates and figures which adorn his contributions to science are by far the best illustrations of Arachnids that have ever been produced in America.” 

His meticuolusly rendered *Illustration of ticks (Ixodida),* illustrates the appendages, forms, and features of 3 specimens accompanyed by his up-close depictions of the capitulum, maxillae, mandibles, stigma, and Haller’s olfactory organ. One can imagine Marx peering through his microscopes and magnifying glasses, methodically rendering and notating his observations with pen and ink, then later refining his sketches for the finished composite. 

In 1896, the year after Marx’s death, Rocky Mountain spotted fever was identified in the Snake River Valley of Idaho, and in 1899, it was first described in a paper by E.E. Maxey. For a decade, its cause eluded researchers until a team led by Dr. Howard T. Ricketts discovered ticks’ role in transmitting Rocky Mountain spotted fever to humans. Eisen and Paddock note that after the bacterium now known as Rickettsia rickettsii was discovered, “18 additional tickborne human pathogens have been recognized; remarkably, more than 40% of these agents have been described since 1980.”

Tickborne viral diseases include Bourbon virus disease, Colorado tick fever, Crimean-Congo hemorrhagic fever, Heartland virus disease, Kyasanur Forest disease, and Powassan encephalitis. Among the tickborne bacterial diseases are anaplasmosis, bartonellosis, ehrlichiosis, Lyme disease, Rocky Mountain spotted fever, and tickborne relapsing fever. Microscopic parasites transmitted by ticks cause the disease babesiosis. The long list of diseases that ticks transmit to humans, none of which were known when Marx created his illustration, and their global incidence continue to increase.
